# Genome‐wide SNPs of vegetable leafminer, *Liriomyza sativae*: Insights into the recent Australian invasion

**DOI:** 10.1111/eva.13430

**Published:** 2022-06-28

**Authors:** Xuefen Xu, Thomas L. Schmidt, Jiaxin Liang, Peter M. Ridland, Jessica Chung, Qiong Yang, Moshe E. Jasper, Paul A. Umina, Wanxue Liu, Ary A. Hoffmann

**Affiliations:** ^1^ Bio21 Institute, School of BioSciences The University of Melbourne Parkville Victoria Australia; ^2^ Cesar Australia Brunswick Victoria Australia; ^3^ Chinese Academy of Agricultural Sciences Beijing China

**Keywords:** biological invasions, ddRAD, gene flow, genome‐wide SNPs, leafminers, population genetics

## Abstract

*Liriomyza sativae*, the vegetable leafminer, is an important agricultural pest originally from the Americas, which has now colonized all continents except Antarctica. In 2015, *L. sativae* arrived on the Australian mainland and established on the Cape York Peninsula in the northeast of the country near the Torres Strait, which provides a possible pathway for pests to enter Australia and evade biosecurity efforts. Here, we assessed genetic variation in *L. sativae* based on genome‐wide single nucleotide polymorphisms (SNPs) generated by double digest restriction‐site‐associated DNA sequencing (ddRAD‐seq), aiming to uncover the potential origin(s) of this pest in Australia and contribute to reconstructing its global invasion history. Our fineRADstructure results and principal component analysis suggest Australian mainland populations were genetically close to populations from the Torres Strait, whereas populations from Asia, Africa, and Papua New Guinea (PNG) were more distantly related. Hawaiian populations were genetically distinct from all other populations of *L. sativae* included in our study. Admixture analyses further revealed that *L. sativae* from the Torres Strait may have genetic variation originating from multiple sources including Indonesia and PNG, and which has now spread to the Australian mainland. The *L. sativae* lineages from Asia and Africa appear closely related. Isolation‐by‐distance (IBD) was found at a broad global scale, but not within small regions, suggesting that human‐mediated factors likely contribute to the local spread of this pest. Overall, our findings suggest that an exotic *Liriomyza* pest invaded Australia through the Indo‐Papuan conduit, highlighting the importance of biosecurity programs aimed at restricting the movement of pests and diseases through this corridor.

## 1 INTRODUCTION

Over the past 40 years, several polyphagous *Liriomyza* (Diptera: Agromyzidae) leaf‐mining species have become recognized as pests capable of causing frequent and severe outbreaks in agricultural commodities (Murphy & LaSalle, 1999). *Liriomyza sativae* Blanchard is one of these leaf‐mining pests, with damage resulting from tunneling activities of larvae that in turn decreases the vigor and the photosynthetic capacity of host plants (Johnson et al., 1983; Parrella, 1987). Leaf punctures caused by female flies of this species also facilitate the transfer of plant disease, with studies demonstrating that female *L. sativae* can transmit viruses of the potyvirus group (Zitter & Tsai, 1977). *Liriomyza sativae* is highly polyphagous across plant families, including Asteraceae, Cucurbitaceae, Fabaceae, Solanaceae, and Umbelliferae (Spencer, 1973), with its host range potentially expanding as it colonizes new areas, as in the case of an expansion to green onions in Hawaii (Carolina & Johnson, 1992).

Although *L. sativae* originated in the Americas, it has now expanded its geographic range and colonized many areas throughout the globe (Scheffer & Lewis, 2005). The movement of infested plant material through international trade and transportation has likely facilitated this process (Minkenberg, 1988). Eggs and larvae of *L. sativae* are embedded internally within plant leaves and can be easily moved from production areas to market without being noticed. The pupae of *L. sativae* may also be transported through infested soil or plant debris, while strong winds facilitate long‐distance adult dispersal (Fenoglio et al., 2019). *Liriomyza sativae* are typical secondary pests with outbreaks occurring after the indiscriminate use of broad‐spectrum insecticides, which removes natural enemies as well as promoting the evolution of resistance (Mason et al., 1987; Oatman & Kennedy, 1976; Parrella & Keil, 1984; Reitz et al., 2013; Ridland et al., 2020). Once *L. sativae* invade new regions, it is difficult to eradicate (Scheffer & Lewis, 2005), highlighting the importance of industry preparedness and biosecurity measures.

Following invasion and establishment into new regions, *L. sativae* often has an immediate and detrimental impact on local horticultural industries (Murphy & LaSalle, 1999). For example, *L. sativae* led to substantial damage in vegetable crops in China after invading in 1993 and expanding into most agricultural areas, with over 2.7 million hectares affected by the end of 2005 at a cost of around 3 billion yuan annually in lost production (Chunlin et al., 2005). Similarly, *L. sativae* was the major pest attacking the foliage of commercial watermelon in Hawaii with an infestation rate of up to 70% of plants (Johnson, 1987) and caused substantial damage to cucumber crops in greenhouses in Iran (Alaei Verki et al., 2020).

*Liriomyza sativae* was first detected in Australia in 2008 when established populations were identified in the Torres Strait (Blacket et al., 2015). Subsequently, *L. sativae* was recorded at Seisia on the northern tip of the Cape York Peninsula, located in the northeastern part of the Australian mainland in 2015 (IPPC, 2017). It is possible this pest arrived through the “Indo‐Papuan conduit,” which covers lands and waterways, connecting southeastern Indonesia and New Guinea with Australia's Torres Strait and Cape York Peninsula (Horwood et al., 2018). This region is known to provide a pathway for the movement of exotic pests and diseases into Australia (Horwood et al., 2018; Thompson et al., 2003). Within Australia, modeling suggests that further spread of *L. sativae* is likely on the mainland unless this can be prevented by quarantine measures (Maino et al., 2019). Pest activity is expected to overlap with the production cycle in Australia of several high‐risk horticultural crops in the vegetable, production nursery, and melon industries (Maino et al., 2019).

Understanding the invasion history of pests like *L. sativae* can help inform quarantine and management strategies, including the likelihood of insecticide resistance genes entering colonizing populations (Ma et al., 2007). The invasion history of a pest can be determined not only from historical records but also through genetic approaches. To date, information on the genetic structure of *L. sativae* populations is limited. Previous genetic research on this species has mainly focused on mitochondrial DNA (mtDNA) for deciphering population structure (Blacket et al., 2015; Parish et al., 2017; Scheffer & Lewis, 2005; Tang et al., 2016; Xu, Coquilleau et al., 2021). While mtDNA markers provide some indication of the relatedness of populations, they have a relatively low resolution particularly in detecting fine‐scale structure (Anderson et al., 2010). Patterns of population relatedness based on mtDNA can also be obfuscated in insects by processes like endosymbiont invasions and recombination across the mitochondrial genomes (Ballard & Whitlock, 2004).

With the increasing ease and speed of DNA sequencing, high‐resolution genomic SNP‐based methods like double digest restriction‐site‐associated DNA sequencing (ddRAD‐seq) provide a new dimension to population‐level studies (Peterson et al., 2012). These markers have been successfully applied to understand patterns of movement in insects that act as pests or disease vectors (Ryan et al., 2019; Schmidt et al., 2019; Yan et al., 2021). They provide a much higher level of resolution than other nuclear marker systems like DNA microsatellites given the sheer number of marker loci that can be scored (Morin et al., 2004). An example of the resolution of population patterns based on SNPs versus microsatellites is provided by Rašić et al. (2014) who showed overlap between populations of the mosquito *Aedes aegypti* based on 8 microsatellite markers but clearly distinct populations when 2300 SNP markers were used. SNP markers scattered across the genome also provide the opportunity to use genetic approaches in tackling new questions such as estimating cross‐generation movement rates of pests following SNP‐based identification of related individuals (Cordeiro et al., 2019; Jasper et al., 2021) and the identification of genomic regions that may be under strong selection once candidate loci have been identified (Endersby‐Harshman et al., 2020; Uchibori‐Asano et al., 2019; Yang et al., 2020).

In this study, we used SNP‐based methods to investigate in detail the incursion patterns of *L. sativae* into the Cape York Peninsula region in Australia. A large panel of SNPs were generated by ddRAD‐seq and screened across 24 *L. sativae* populations, focusing mostly on the Asia‐Pacific region but also including some worldwide samples. This study aimed to: (a) explore the population genetic structure of *L. sativae* across this region, (b) determine the possible source(s) of the recent incursions in Australia, and (c) provide a baseline for future explorations of further *L. sativae* incursions across the Australian mainland.

## 2 MATERIALS AND METHODS

### 2.1 Sample collections, DNA extraction, and DNA barcoding

**FIGURE 1:**
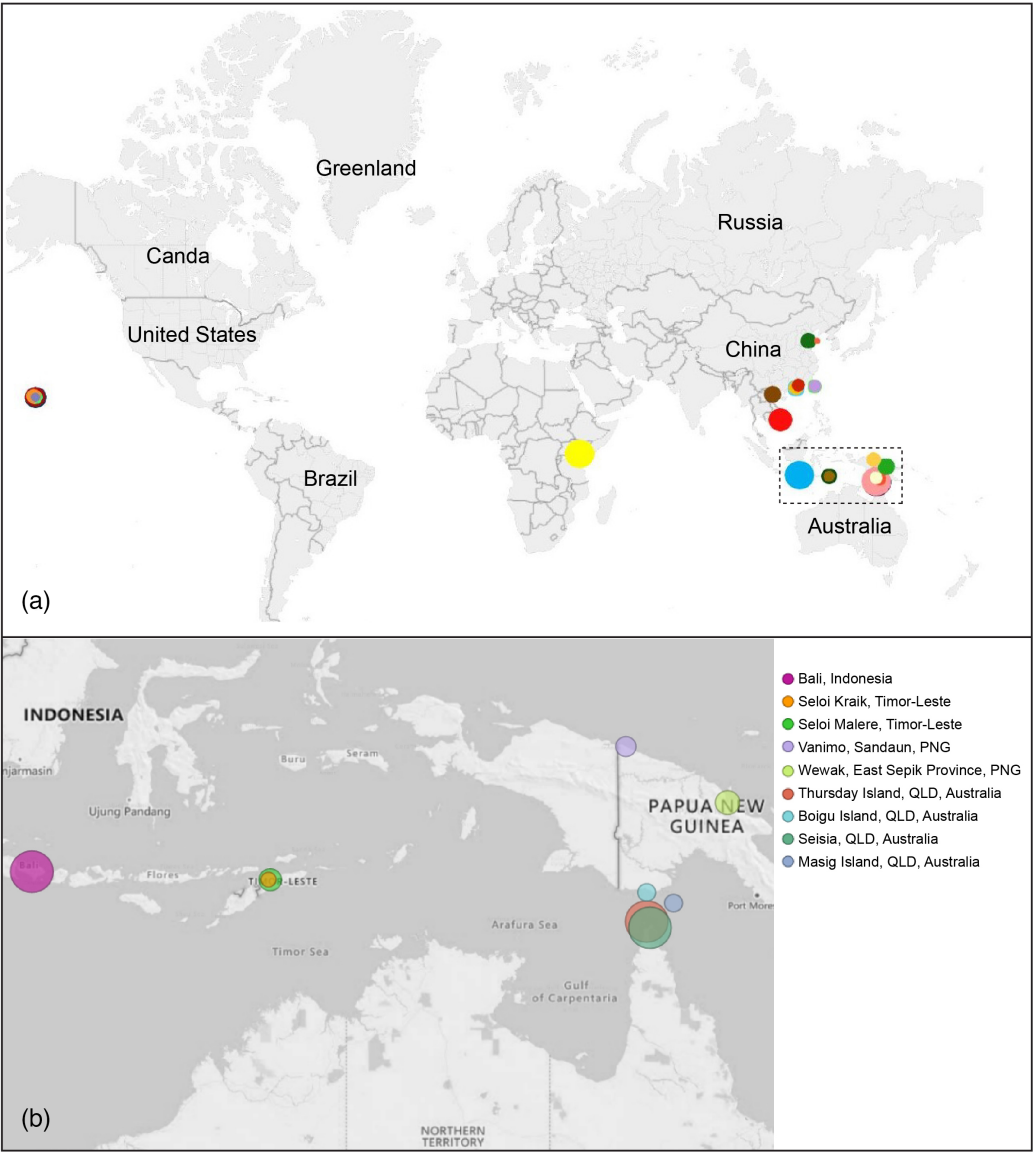
(a) Sampling locations of *Liriomyza sativae* in this study. Colors indicate different populations, and the size of circles reflects the relative sizes of each sample. The dotted rectangle represents the local geography around northern Australia, as detailed in (b). QLD = Queensland; PNG = Papua New Guinea

*Liriomyza sativae* field samples were obtained from 24 populations from eight countries between July 2016 and May 2020 through active sampling (Figure 1, Table 1), which depended on the goodwill and time availability of many collaborators. Australian specimens were collected from three Torres Strait Islands (Boigu Island, Masig Island, and Thursday Island) and from one mainland location (Seisia). Samples were preserved in 100% ethanol at −80°C until DNA extraction. As species of *Liriomyza* have similar external morphology, taxonomic identification of *L. sativae* samples was confirmed by both morphological identification (by Dr. Mallik Malipatil, Agriculture Victoria, AgriBio, La Trobe University, Bundoora, Australia) and barcodes based on mitochondrial cytochrome c oxidase subunit I (CO1). For barcoding, legs of flies were taken from specimens for Chelex DNA extraction with protocols following previous work except in 70 μl of 5% Chelex 100 resin (Bio‐Rad Laboratories, Gladesville, NSW, Australia) (Coquilleau et al., 2021; Xu, Coquilleau et al., 2021; Xu, Ridland et al., 2021). The criteria for assigning CO1 haplotypes were based on BLAST similarity in NCBI (Johnson et al., 2008) with 100% similarity being used to link to existing haplotypes, and assignment to *L. sativae* clades defined by a similarity threshold of <2.5% (Scheffer & Lewis, 2005). Once species identity was confirmed, whole fly bodies were used for ddRAD‐seq libraries.

**TABLE 1:** Collection details for the 193 *Liriomyza sativae* samples retained after quality filtering for ddRAD sequencing

Collection date	Country	Location	Latitude	Longitude	Host plant	*n*
2017.07	Australia	Seisia, QLD	−10.850	142.368	*Macroptilium atropurpureum*	20
2018.07	Australia	Thursday Island, QLD	−10.575	142.221	*Macroptilium atropurpureum*	20
2019.06	Australia	Masig Island, QLD	−9.751	143.408	*Ricinus communis*	4
2019.06	Australia	Boigu Island, QLD	−9.274	142.223	*Macroptilium atropurpureum*	4
2019.11	Papua New Guinea	Vanimo, Sandaun,	−2.685	141.304	*Cucumis sativus*	5
2017.11	Papua New Guinea	Wewak, East Sepik Province	−5.225	145.793	*Solanum lycopersicum*	7
2018.11	Timor‐Leste	Seloi Malere	−8.704	125.609	*Cucurbita pepo*	6
2017.09	Timor‐Leste	Seloi Kraik	−8.698	125.534	*Cucurbita pepo*	3
2019.04	Indonesia	Bali	−8.340	115.091	*Cucumis sativus*	20
2020.01	Vietnam	Lien Kiep	11.739	108.360	*Benincasa hispida*	13
2020.01	Vietnam	Sao Bay	20.592	105.601	*Cucumis sativus*	7
2016.07	China	Shenzhen, Guangdong	22.664	113.888	*Luffa acutangula*	7
2016.07	China	Dongguan, Guangdong	22.923	113.899	*Vigna sinensis*	5
2017.12	China	Heyuan, Guangdong	23.716	114.681	*Solanum lycopersicum*	4
2016.08	China	Dongying, Shandong	37.564	118.246	*Cucurbita moschata*	6
2016.09	China	Yantai, Shandong	37.504	121.369	*Phaseolus vulgaris*	1
2019.09	China	Chiayi, Taiwan	23.400	120.551	*Luffa cylindrica*	4
2019.09	China	Tainan, Taiwan	23.283	120.447	*Luffa cylindrica*	5
2019.05	USA	Keaau, Hawaii	19.629	−155.027	*Solanum nigrum*	2
2019.05	USA	Paradise Park, Hawaii	19.588	−154.96	*Solanum melongena*	11
2019.05	USA	USDA‐ARS‐PBARC, Hawaii	19.698	−155.093	*Solanum melongena*	9
2019.05	USA	Kawamata Farms, Hawaii	20.011	−155.684	*Solanum melongena*	5
2019.05	USA	Hilo, Hawaii	19.671	−155.100	*Solanum melongena*	5
2020.01	Kenya	Kirinyaga County	−0.615	37.375	*Solanum lycopersicum*	20

Abbreviation: QLD, Queensland.

### 2.2 ddRAD‐seq library preparation

We built three ddRAD libraries for 200 *L. sativae*, each containing between 40 and 80 individuals. Genomic DNA from individual *L. sativae* was extracted with a DNeasy Blood and Tissue kit (Qiagen, Venlo, Limburg, NL). ddRAD‐seq libraries were prepared according to the protocol from Rašić et al. (2014). In brief, around 45 ng of high‐quality genomic DNA was simultaneously double‐digested using restriction enzymes *MluC*I (10 units; New England Biolabs, Beverly MA, USA) and *Nla*III (10 units; New England Biolabs), NEB CutSmart buffer, and water. Digestions were incubated at 37°C for 3 h, and then the products were cleaned with 1.5× volume of AMpure XP paramagnetic beads (Beckman Coulter, Brea, CA, USA). After that, the digestion fragments were ligated to modified adapters containing 6 bp indexes at both ends overnight at 16°C with T4 ligase (1100 units; New England Biolabs), followed by a heat deactivation step at 65°C for 10 min. Adapter ligated DNA fragments from all individuals were pooled and cleaned with 1.5× bead solution afterward. A Pippin Prep cassette of 2% agarose with marker B (Sage Sciences, Beverly, MA, USA) was applied for size selection of fragments between 350–450 bp. Lastly, 1 μl size‐selected DNA was added in a 10 μl PCR as a template with 5 μl of the Phusion High Fidelity 2× Master mix (New England Biolabs) and 2 μl of 10 μM Illumina PCR primers. PCR programs were run at 98°C for 30 s, followed by 15 cycles of 98°C for 10 s, 60°C for 30 s, 72°C for 90 s, and a final elongation step at 72°C for 5 min. We pooled 10 such PCR products together and cleaned them with a 1.5× bead solution for making the final library. The ddRAD‐seq libraries were sequenced on an Illumina NovaSeq 6000 platform using 2 × 150 bp chemistry by Novogene HK Company Limited, Hong Kong.

### 2.3 Sequence data processing and genotyping

We processed the raw sequencing reads according to the STACKS v2.54 pipeline (Catchen et al., 2013). Quality filtering of raw reads was performed with the process_radtags program. This program first checks if the barcode and the RAD cut site are intact, and then demultiplexes the data, correcting errors within allowable limits. We first trimmed the reads to 115 bp in length (‐t 115) and removed sequence reads with average Phred scores below 20 (‐s 20), a cutoff used in similar studies (Chen et al., 2021; Schmidt et al., 2019). Given the absence of a reference genome for *L. sativae*, we used the Stacks workflow (ustacks, cstacks, sstacks, tsv2bam, and gstacks) to build a catalog of loci de novo (Catchen et al., 2011). In brief, we first processed all samples with ustacks (‐m 3 ‐M 4) and then selected a subset of the 200 samples (including a few individuals from each population) to build a catalog with cstacks (‐n 4). Then, using the cstacks catalog, all 200 samples were run through sstacks and the rest of the steps in the denovo_map workflow. The final SNP data set included all the markers observed in a tag. After catalog construction, we removed seven samples (i.e., leaving *n* = 193) due to a high level of missing data (>50%) and generated VCF files containing SNPs called in 75% of individuals (‐r 0.75) from each population (‐p 24) with a minor allele count of 3 (‐‐min‐mac 3) for downstream analyses (*c.f*. Hoffmann et al., 2021).

### 2.4 Population structure

#### 2.4.1 Principal component analysis

We undertook a principal component analysis (PCA), by using the “prcomp” function (scale = TRUE, in order to scale the variables to unit variance) implemented in the ade4 R package (Dray & Dufour, 2007). We ran a PCA with 7379 SNPs to summarize genetic variation across all populations.

#### 2.4.2 fineRADstructure analysis

We performed a co‐ancestry analysis on all *L. sativae* (193 individuals) with fineRADstructure (Malinsky et al., 2018), which is designed to infer recent population structure (available at https://github.com/millanek/fineRADstructure). The co‐ancestry matrix of 193 individuals was based on a summary of nearest neighbor haplotype relationships, which were calculated from multiple SNPs per RAD locus. The individuals were assigned to corresponding populations, and a phylogenetic tree was constructed using the FineRADstructure MCMC clustering algorithm with default settings. R scripts fineradstructureplot.r and finestructurelibrary.r (available at http://cichlid.gurdon.cam.ac.uk/fineRADstructure.html) were used for visualization.

#### 2.4.3 Admixture analysis

Admixture v.1.3.0 (Alexander et al., 2009) was used to infer genetic structure assuming a specific number of ancestral lineages (*K*). This tool is based on a maximum likelihood method to estimate individual ancestries, and a cross‐validation (CV) test was applied to evaluate the optimal K (Alexander & Lange, 2011). To avoid stochastic effects from a single analysis, we ran 10 iterations at each value of *K*. Data were visualized using the ComplexHeatmap and viridis packages in R (downloaded packages from https://www.bioconductor.org/).

#### 2.4.4 Isolation‐by‐distance

To test whether genetic differentiation patterns matched expectations from isolation‐by‐distance (IBD), we used a Mantel test to explore the correlation between genetic distances (*F*_ST_) and geographic distances (km) based on 999 permutations implemented in adegenet 2.1 (Jombart, 2008). To avoid sampling bias, we removed populations with fewer than four individuals (Yantai, China; Keaau, USA; and Seloi Kraik, Timor‐Leste), leaving 21 populations for the overall IBD analysis. We also ran a region‐specific Mantel test that included eight populations of particular interest to the recent *L. sativae* invasion into Australia; these were Bali, Seloi Malere, Vanimo, Wewak, Boigu Island, Masig Island, Thursday Island, and Seisia (Table 1). Genetic divergence was estimated by calculating F_ST_ values between all pairs of populations using the “genepop” R package (Rousset et al., 2020). The geosphere R package v.1.5‐5 was applied to calculate straight‐line geographic distances between populations (Hijmans et al., 2017). *p*‐Values smaller than 0.05 were considered significant.

## 3 RESULTS

### 3.1 Principal component analysis

**FIGURE 2:**
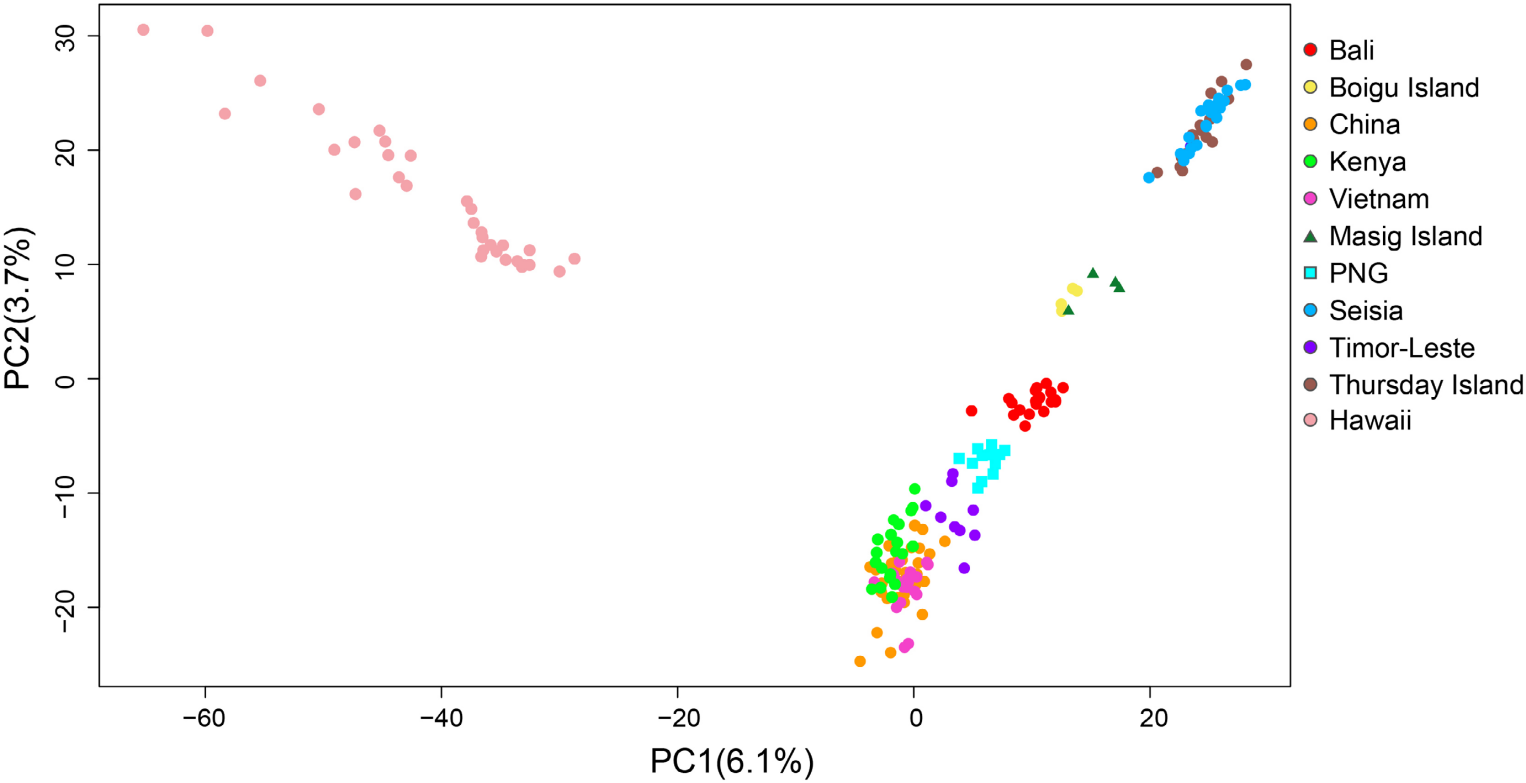
Principal component analysis (PCA) of 193 *Liriomyza sativae* individuals collected from 24 populations based on 7379 SNPs. The variances explained by PC1 and PC2 are given. Each dot represents sampled individuals, with populations from the same country merged except for the Australian populations (Boigu Island, Masig Island, Seisia, and Thursday Island). PNG = Papua New Guinea

Genetic structure among *L. sativae* individuals across all populations was visualized using a PCA incorporating 193 individuals and 7379 SNP loci (Figure 2). There was a strong separation of Hawaii from all other populations, with the main axes accounting for 6.1% and 3.7% of the variation.

### 3.2 fineRADstructure analysis

**FIGURE 3:**
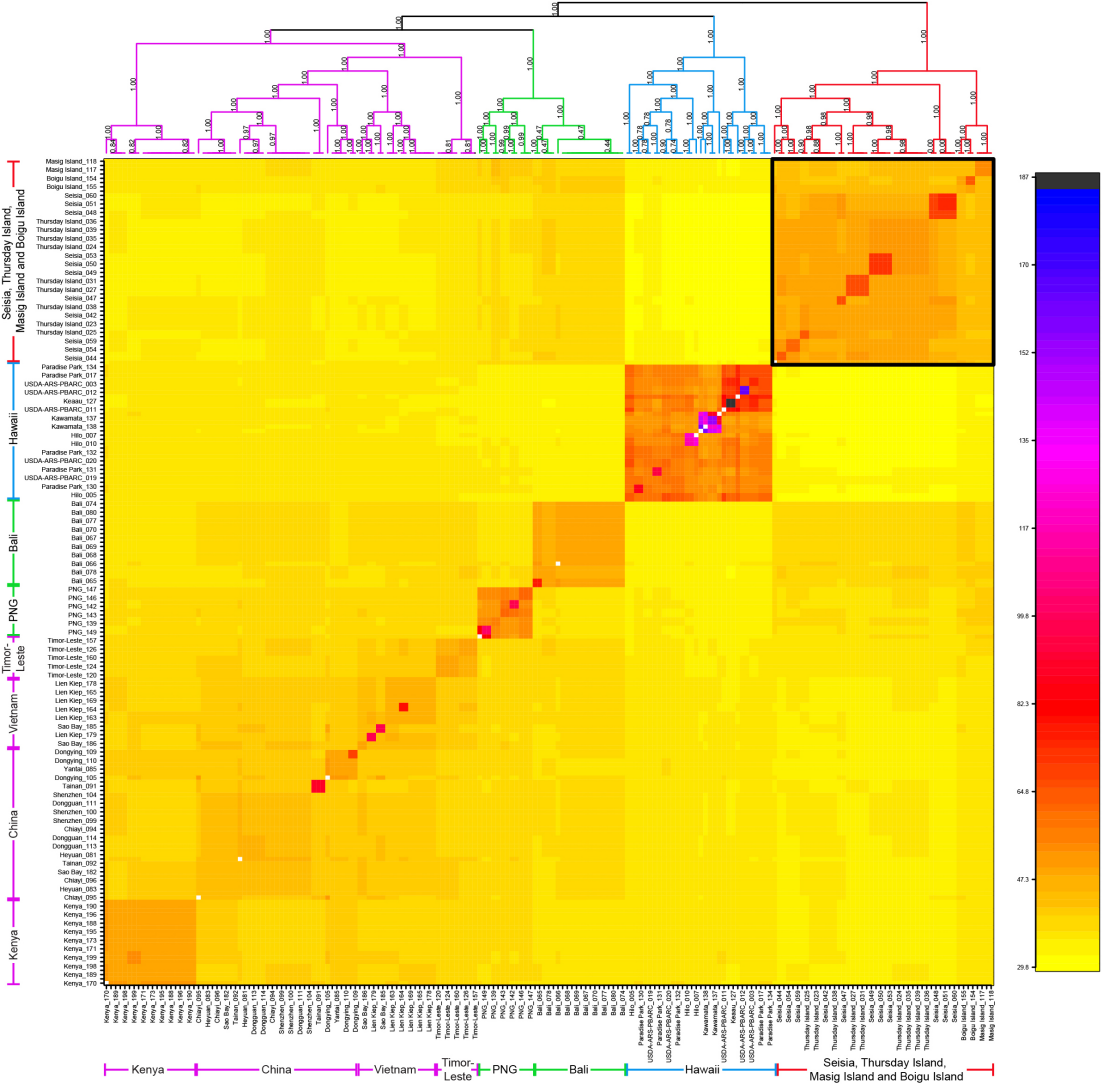
FineRADstructure plot with co‐ancestry map and phylogenetic tree. The color scale bar indicates estimated co‐ancestry, with light yellow suggesting low co‐ancestry and darker yellow, orange, and red indicating progressively higher co‐ancestry. The Australian populations formed a clade (labeled in red), which is shown in the solid black square. See Figure S1 for full‐size figure

The fineRADstructure plot (Figure 3) shows four subgroups among selected locations. The biggest subgroup placed Asian individuals from China and Vietnam together with Timor‐Leste and the single African population from Kenya. The genotypes of Bali formed a lineage with PNG that was close to the Asia/Africa clade. However, *L. sativae* from PNG were genetically distinct from Bali, suggesting these regions share some recent co‐ancestry but can still be differentiated using fineRADstructure analysis. Genotypes from the Hawaii populations cluster together but with a high level of differentiation between them. The Hawaiian lineage was strongly differentiated from the other lineages, indicating an invasion history of *L. sativae* in Hawaii separate from the other populations. Torres Strait populations formed a lineage with the Australian mainland population from Seisia, suggesting recent co‐ancestry among these populations (Figure S1). The phylogenetic tree showed that the individuals of Thursday Island and Seisia are mixed together and independent from groups of Masig Island and Boigu Island, and there is no structure between them. This analysis also revealed that the Australian mainland was likely invaded from the Torres Strait instead of other regions. Interestingly, the Australian populations were most closely related to Bali, followed by PNG, suggesting that Bali and PNG were important sources for the initial invasion of *L. sativae* into the Torres Strait Islands.

### 3.3 Admixture analysis

**FIGURE 4:**
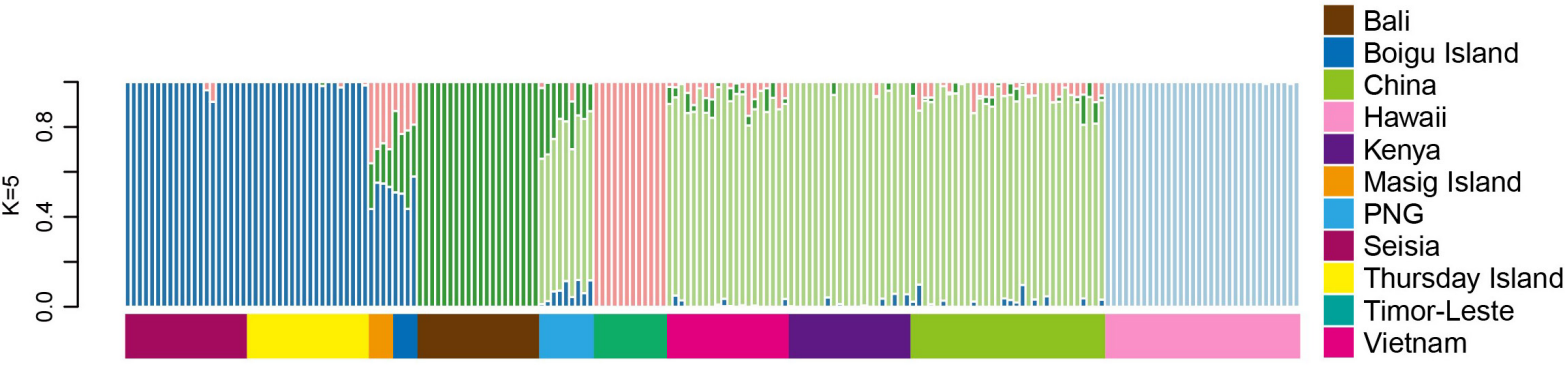
Population structure of *Liriomyza sativae* based on 193 individuals inferred using admixture analysis. The minimum cross‐validation error was found when *K* = 5. PNG = Papua New Guinea

To infer the genetic structure and the degree of relatedness among *L. sativae* populations with different genetic backgrounds, we ran an admixture analysis with cross‐validation for values of *K* (subgroups) from 2 through 10 to examine patterns of ancestry based on the 193 individuals (Figure S2). Note that a plot with *K* = 1 was not considered given the low likelihood of such a scenario based on existing literature (Yamashita et al., 2019). A 10 fold cross‐validation (CV) procedure was performed to infer the optimal number of subgroups. We found a minimum CV score was obtained when *K* = 5, and we used this value in an admixture plot (Figure 4). When *K* = 5, individuals from Hawaii formed a distinct cluster, which was also supported in the admixture plots using different *K* values (*K* = 2–4) (Figure S3). Individuals from Vietnam, Kenya, and China share the same ancestries and cluster together, suggesting these populations have been derived from a common origin. Timor‐Leste shares a similar ancestry with Asia/Africa and, to a lesser extent, with individuals from Bali and Australia. Bali and PNG individuals represent different genetic lineages, with a low proportion of putative admixture pointing to separate introduction events.

Interestingly, the admixture analysis suggests Bali and PNG contributed most to the ancestries of two (of the three) Torres Strait populations: Boigu Island and Masig Island. New genotypes were detected leading to novel ancestry for Thursday Island and Seisia. Admixture results also indicate recent co‐ancestry from the Torres Strait to mainland Australia. Compared with Seisia, *L. sativae* from Boigu Island and Masig Island contained higher levels of genetic diversity (including new genotypes) that are likely associated with multiple introduction sources (e.g., Bali, PNG, and potentially Timor‐Leste).

When visualizing the population structure when *K* = 8 (the second‐best *K* value), there are additional hierarchical structure levels, primarily involving Kenya, but also within Australian *L. sativae* populations, and within Hawaii (Figure S3).

### 3.4 Isolation‐by‐distance

To evaluate the possible association between genetic and geographic distances, we built matrices of genetic distances among 21 populations (excluding three populations with sample size less than four) by calculating pairwise *F*_ST_ values and then correlating these with pairwise geographic distances between populations based on 3318 SNPs. Pairwise *F*_ST_ values among all populations ranged from 0.0045 to 0.3269 (Table S1). Relatively stronger differentiation was observed between Kawamata Farms (Hawaii, USA) and other locations (*F*_ST_ values ranging from 0.148 to 0.3269). The highest *F*_ST_ value (0.3269) was between Kawamata Farms (Hawaii, USA) and Seisia (QLD, Australia). On the contrary, the lowest *F*_ST_ value (0.0045) was between Heyuan (Guangdong, China) and Chiayi (Taiwan, China). Pairwise *F*_ST_ values within Hawaiian populations ranged from 0.018 to 0.177, suggesting high genetic diversity within Hawaii. Specifically, the pairwise *F*_ST_ values between Paradise Park (Hawaii, USA), Hilo (Hawaii, USA), and USDA‐ARS‐PBARC (Hawaii, USA) ranged from 0.0181 to 0.0502, while the pairwise *F*_ST_ values between Kawamata Farms (Hawaii, USA) and all other Hawaiian populations ranged from 0.148 to 0.1775.

**FIGURE 5:**
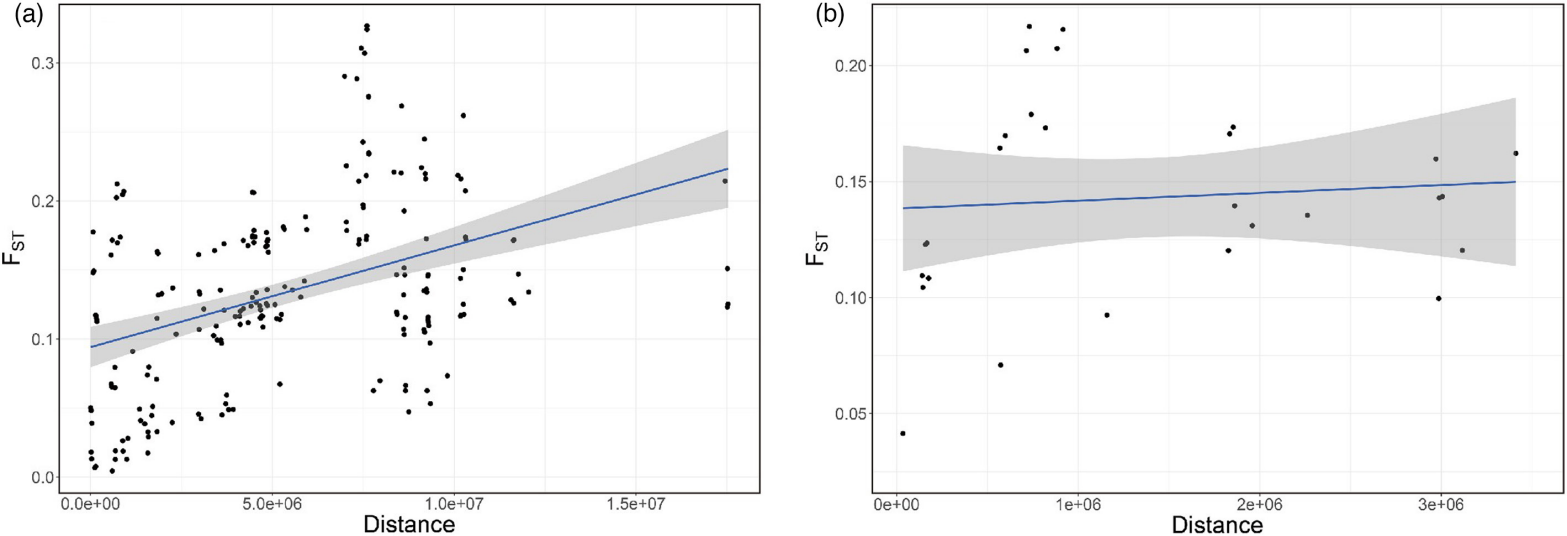
(a) Association between genetic distances (*F*_ST_) and geographic distances (km) for the 21 *Liriomyza sativae* populations (Mantel test, *p* = 0.002; correlation = 0.412; *R* ^2^ = 0.166). (b) Association between genetic distances (*F*_ST_) and geographic distances (km) for the 8 *L. sativae* populations associated with the Australian incursion (Mantel test, *p* = 0.216). The line in the picture is a linear model fitted to the data, and the shading represents the associated 95% confidence interval

At a large spatial scale (across the 21 populations), we found support for isolation‐by‐distance, with a Mantel test being significant (*p* = 0.002; correlation = 0.412; *R* ^2^ = 0.166) based on 999 replicates (Figure 5a). IBD results revealed a significant positive correlation between the geographic and genetic matrices in the large‐scale comparison. We also examined IBD using populations that might be involved in the recent Australian *L. sativae* incursion (Bali, Seloi Malere, Vanimo, Wewak, Boigu Island, Masig Island, Thursday Island, and Seisia). Pairwise *F*_ST_ between these populations ranged from 0.0414 to 0.2169 (Table S2). Relatively stronger differentiation occurred between the two populations in PNG (Vanimo and Wewak) and the remaining populations (*F*_ST_ values ranging from 0.1355 to 0.2169). The highest *F*_ST_ value (0.2169) was between Wewak and Seisia (QLD, Australia). On the contrary, the lowest *F*_ST_ value (0.0414) was between Thursday Island (QLD, Australia) and Seisia. A Mantel test indicated no relationship between pairwise genetic (*F*_ST_) and geographic distances (*p* = 0.241 based on 999 replicates) (Figure 5b), suggesting that distance isolation had a low effect on genetic differentiation at this smaller spatial scale.

## 4 DISCUSSION

With the increasing globalization of trade and an escalating volume of goods being moved, threats posed by invasive pest species are increasing (Hulme, 2009). *Liriomyza sativae* represents one such threat, having invaded many countries around the world in the past 40 years (Murphy & LaSalle, 1999; Scheffer & Lewis, 2005). In Australia, *L. sativae* has now established in the northeastern part of the mainland and could expand into other regions (Maino et al., 2019). Here, we conducted the first population genetic analyses of *L. sativae* individuals based on genome‐wide SNPs, aiming to trace incursion pathways into the Australian mainland and around the Indo‐Papuan region.

We found expected signals of gene flow among *L. sativae* populations from the Torres Strait into the Australian mainland. The allelic composition of the Thursday Island (Torres Strait) population is nearly identical to that from Seisia (mainland Australia). However, these two populations are genetically different from Masig Island and Boigu Island (both from the Torres Strait), although all these populations still cluster together as a group when compared to other localities. Our study, therefore, provides evidence that the incursion of *L. sativae* into mainland Australia is directly linked to Torres Strait populations rather than arising from incursions from other countries. We also found support for multiple invasions from different countries (especially Indonesia, PNG, and Timor‐Leste) into the Torres Strait populations as reflected by results from the admixture analyses. Furthermore, we found that *L. sativae* populations in Hawaii are genetically distinct from other countries, whereas samples from Kenya, China, Vietnam, and Timor‐Leste cluster as a group that is related to Bali and PNG populations.

*Liriomyza sativae* was first described from specimens collected from alfalfa (*Medicago sativa*) in Argentina in 1937 (Blanchard, 1938), although a South American origin for the species has not been validated with genomic markers. *Liriomyza sativae* (incorrectly named *Agromyza pusilla)* was reported to be damaging crops in mainland USA before World War 1 (Spencer, 1973). By 1980, *L. sativae* was common in southeast USA, Central America, and northern parts of South America (Hardy & Delfinado, 1980). The earliest recorded collection of *L. sativae* in Hawaii was in 1921 (Table S3), where it was described as *Liriomyza canomarginis*, but later synonymized with *L. sativae* (Spencer, 1973; Table S3). Given Hawaii imports most of its vegetables from mainland USA (Parcon et al., 2010), imported produce is the likely source of incursions. The CO1 haplotype of *L. sativae* in Hawaii belongs to the *L. sativae*‐W clade (Xu, Coquilleau et al., 2021), which is also widespread in North America, having been collected from crops in California, Florida, and Arizona (Scheffer & Lewis, 2005). In contrast, *L. sativae*‐A clade is common in Florida and has not been recorded in Hawaii, suggesting that *L. sativae* in Hawaii has a west coast origin. The *L. sativae*‐W clade is the invasive clade, whereas all other clades are found in the Americas. Apart from highlighting the genetic uniqueness of the populations from Hawaii, our data also indicate strong differentiation between the northern (Kawamata Farms) and eastern (remaining) groups. Unfortunately, our study did not have samples from the suspected native range of South America as a reference. Populations of *L. sativae* from Hawaii might be strongly differentiated from mainland USA due to genetic drift, although this might also reflect repeated ongoing introductions from the mainland.

The movement of *L. sativae* to Hawaii (first recorded in 1921) and then to several South Pacific islands (Waterhouse & Norris, 1987) occurred at least 20 years before the pest moved through Asia (Martinez, 1994) and Africa (Martinez & Bordat, 1996). In turn, from the mid‐1970s, *Liriomyza trifolii* (Burgess) has largely displaced *L. sativae* in southern parts of the USA (Parrella, 1982) and Hawaii (Johnson, 2005). The spread of *L. sativae* has speeded up since the 1990s when *L. sativae* moved eastward through tropical and sub‐tropical areas like Africa, China, Indonesia, and Vietnam (Andersen et al., 2002; Chen & Zhao, 1999; Rauf et al., 2000; Ridland et al., 2020). *Liriomyza sativae* arrived in Indonesia in the 1990s and became a major pest in lowland vegetable crops (Rauf et al., 2000). The species was then recorded in Timor‐Leste in 2003 and West Papua in 2005. In 2008, *L. sativae* were first collected on Warraber Island in the Torres Strait in a heavily mined tomato plant (Blacket et al., 2015). Subsequently, it was recorded in the central highlands of PNG in 2011 and other Torres Strait Islands (Blacket et al., 2015). In 2015, *L. sativae* was recorded on mainland Australia at Seisia, at the tip of Cape York (IPPC, 2017). Our admixture results are congruent with this population expansion pattern of *L. sativae* in Australia: an initial introduction in 2008 into the Torres Strait, followed by a rapid expansion over multiple islands before invading the northern Cape York Peninsula area in 2015. In Kenya, *L. sativae* had become a common pest by the early 2000s (Gitonga et al., 2010; Musundire et al., 2011).

Our population genetic results are broadly congruent with the biogeographical history of *L. sativae*. The genotypes in Kenya, China, Vietnam, and Timor‐Leste are genetically related, consistent with a relatively recent invasion across these regions. Genetic data also support a recent invasion of this species across 12 provinces in China with relatively low levels of genetic diversity (Tang et al., 2016). The spread of *L. sativae* across Timor‐Leste was likely caused by multiple introductions; thus, some level of ongoing gene flow was likely during the initial incursion stages. Unfortunately, we lack samples from southeast Asia (e.g., Malaysia and Philippines), which would be required to adequately assess these possibilities. Further sampling in southeast countries near Timor‐Leste may help reveal this part of the incursion history in more detail. Genetic variation in Hawaii, Asia, and Africa was relatively low based on admixture results, which suggests a possible demographic bottleneck at the time of introduction and/or a period of limited population growth after establishment. The pattern of genetic differentiation across these populations indicates a limited contribution to the *L. sativae* expansion in Australia.

Despite these connections between distant locations, IBD was still detected overall, suggesting that geographical distance, wind currents, ocean currents, and obstructing landmasses at a broader scale may have affected genetic connectivity of *L. sativae* populations globally (Ben Abdelkrim et al., 2017; Broquet et al., 2006; White et al., 2010). On the contrary, when a smaller scale was considered (populations related to the recent Australia incursion), genetic distances were not significantly correlated to geographic distances, suggesting that human‐mediated movements may have contributed to local spread.

Although the SNP data provide a detailed picture of relatedness that is not available from mtDNA data, our results are nevertheless consistent with some previous CO1 results. These indicated a link between the Torres Strait Islands and the mainland incursion as well as the possibility of multiple incursions into Torres Strait populations (Blacket et al., 2015; Xu, Coquilleau et al., 2021). Previous CO1 studies demonstrate that S.27 is the dominant haplotype in Indonesian and Australian populations whereas one population (Erub Island) in the Torres Strait possesses haplotype S.07, the dominant haplotype found in PNG (Blacket et al., 2015; Xu, Coquilleau et al., 2021). The SNP data generated in our study provide a much clearer signature of admixture in the Torres Strait populations, including Thursday Island and Seisia which are only separated by 34 km. In future studies, it will be interesting to track further incursions into this area and beyond. Currently, the mainland population at Seisia is under quarantine to prevent the spread of *L. sativae* to other regions, but further spread seems inevitable given that many other regions on the mainland are suitable for this species to exist (Maino et al., 2019). Any further incursions into the Torres Strait may benefit *L. sativae* such as through the introduction of insecticide resistance genes. The geographical location of the Torres Strait is particularly suitable for multiple incursions as detected in species like the Asian tiger mosquito, *Aedes albopictus* (Beebe et al., 2013; Schmidt et al., 2021), island sugarcane planthopper *Eumetopina flavipes* (Anderson & Congdon, 2013), as well as *Culicoides* biting midges (Eagles et al., 2014). Pests can move into the Torres Strait by natural processes (wind currents) as well as human activities (vessel movements and fishing activity) (Kompas et al., 2015).

## 5 CONCLUSIONS

In summary, our SNP‐based analyses of *L. sativae* provide evidence of connections among populations that are mostly consistent with historical observations. Our study is the first to apply high‐density SNP markers to determine the population structure of *Liriomyza* and provide a baseline and foundation to further track leafminer movements across the region and further into the Australian mainland. The data provide a basis for quarantine detections to identify local versus international sources (*c.f*. Schmidt et al., 2019) and an ongoing assessment of new incursions can indicate risks associated with insecticide resistance genes entering local populations.

## CONFLICT OF INTEREST

None declared.

## Supporting information


Appendix S1

## Data Availability

The data that support the findings of this study are freely available from the NCBI BioProject (accession PRJNA838088).
