# Supplementary figures and images for: Genome‐wide SNPs of vegetable leafminer, Liriomyza sativae: Insights into the recent Australian invasion
Source: Evol Appl. 2022 Jun 28;15(7):1129–40. doi: 10.1111/eva.13430 (PMC9309458; doi:10.1111/eva.13430)

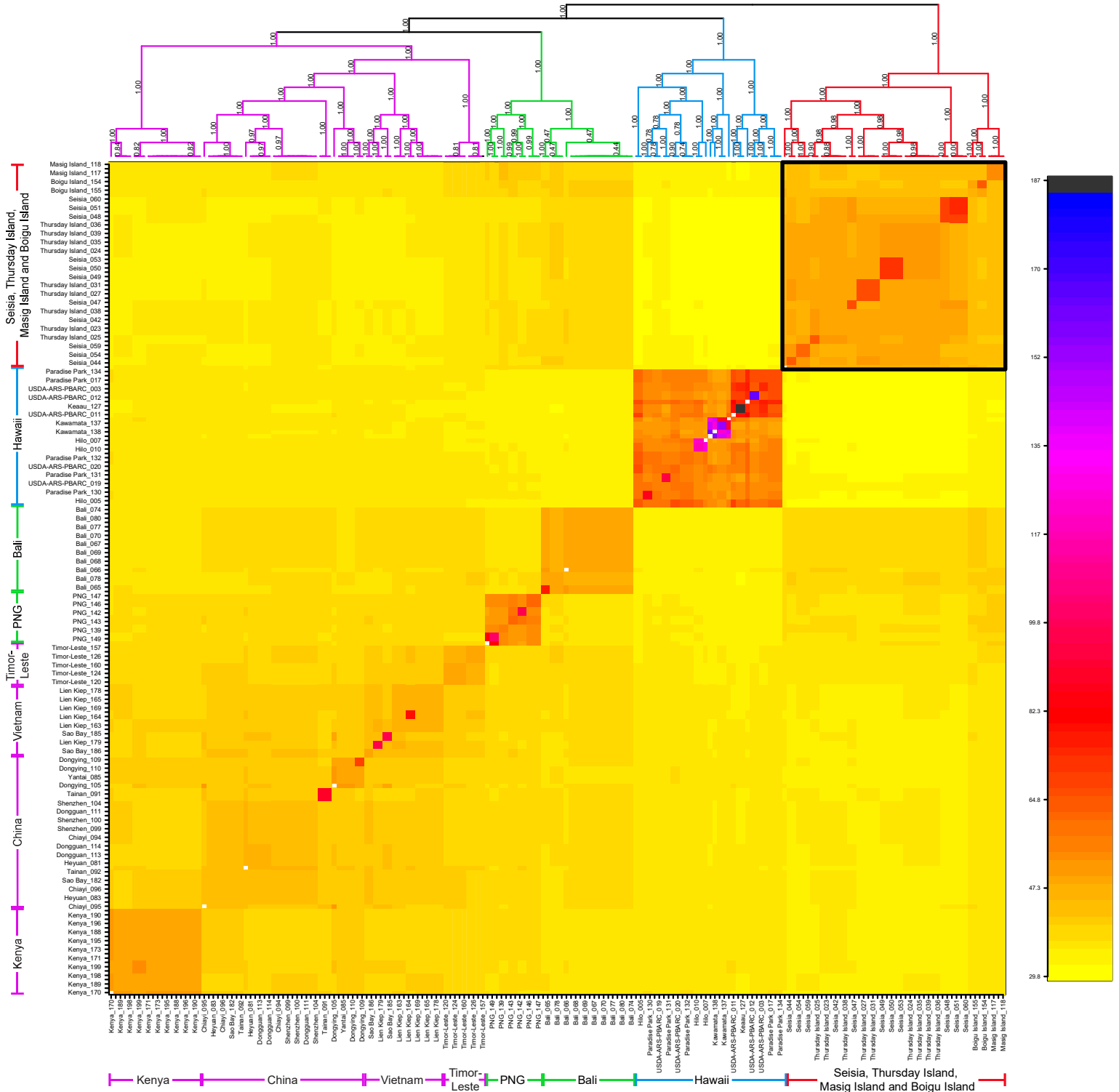

Supplement: Supplementary file 1 — Appendix S1 [file EVA-15-1129-s001.zip › EVA_13430_Hoffmann_Supplementary Information_Fig. S1.pdf]
